# An unusual case of subacute small bowel obstruction - Gallstone ileus

**DOI:** 10.1016/j.ijscr.2022.106820

**Published:** 2022-02-08

**Authors:** Nischal Shrestha, Aakash Mishra, Roshan Ghimire

**Affiliations:** a Department of Surgery, Kathmandu Medical College Teaching Hospital, Kathmandu, Nepal; b Kathmandu Medical College Teaching Hospital, Kathmandu, Nepal

**Keywords:** Gallstone ileus, Cholecystoduodenal fistula, Intestinal obstruction, Enterotomy

## Abstract

**Introduction and importance:**

Gallstone ileus is caused by an impaction of one or more gallstones within the gastrointestinal tract, leading to mechanical intestinal obstruction. It is a rare complication of cholelithiasis leading to the formation of a cholecystoenteric fistula and is associated with high mortality rates. We report a case of atypical subacute small bowel obstruction due to gallstone ileus.

**Presentation of case:**

An 82-year-old man, with previously diagnosed cholelithiasis, presented with abdominal pain and vomiting for nine days. The contracted gallbladder with distended bowel loops was visualized on abdominal ultrasound. Computed tomography of the abdomen and pelvis revealed dilated loops of the small intestine with a gallstone in the proximal ileum, causing intestinal obstruction with pneumobilia, suggesting gallstone ileus with cholecystoduodenal fistula. The patient underwent an emergency laparotomy and enterolithotomy to remove the impacting gallstone. The cholecystoduodenal fistula was left undisturbed due to the significant risk of duodenal injury. The patient had an uneventful postoperative recovery.

**Conclusion:**

Gallstone ileus almost always requires surgical management. However, performing an interval biliary surgery is based on the clinical judgment of the surgeon. In our case, the patient's clinical status determined the treatment in which an enterotomy with stone extraction alone was largely sufficient, and has supported the literature. Gallstone ileus is an important differential diagnosis in elderly patients with gallstone disease, untreated or undiagnosed, presenting with features of small bowel obstruction.

## Highlights

• Gallstone ileus is a rare cause of small bowel obstruction, commonly in the elderly.
• Cholelithiasis, untreated or undiagnosed, may result in a cholecystoduodenal fistula and gallstone ileus.
• CT scan is the preferred imaging modality and characteristic radiological findings aid in early diagnosis.
• An enterotomy with stone extraction is sufficient in comorbid patients without intruding on the cholecystoduodenal fistula.

## 1 Introduction

Gallstone induced small-bowel obstruction is an uncommon complication of a common condition, cholelithiasis. Gallstone ileus (GI) occurs due to gallstone impaction in the gastrointestinal tract, commonly through a cholecystoduodenal fistula [1]. It is associated with relatively high rates of morbidity and mortality [2]. The presented case is one of a gallstone causing subacute small bowel obstruction that was managed by open enterolithotomy. This work has been reported following the SCARE 2020 guidelines [3].

## 2 Case presentation

An 82-year-old frail gentleman presented to our center with a nine-day history of abdominal distension, constipation, localized periumbilical pain, intermittent, nonradiating, and multiple episodes of nonprojectile, nonbilious vomiting containing food particles. He denied fever, jaundice, hematochezia/melena, urine or urine discolored, or trauma to the abdomen before the onset of the symptom. The medical history was significant for hypertension since 15 years, cholelithiasis diagnosed eight years ago, and recently diagnosed bronchiectasis, managed with low flow oxygen therapy.

**Fig. 1:**
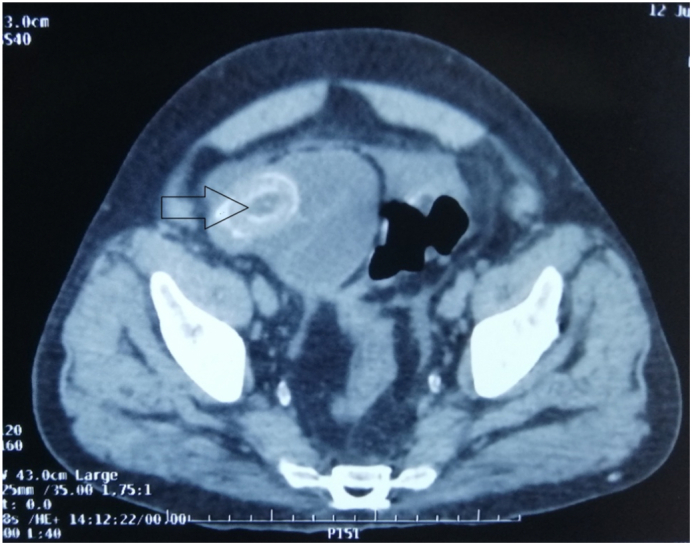
Gallstone (arrow) causing bowel obstruction.

On examination, his vital parameters were within normal limits, abdomen distended, nontender with exaggerated bowel sounds. Initial investigations showed normal hematologic and biochemical parameters. Abdominal ultrasound revealed a contracted gallbladder with distended bowel loops. Computed tomography of the abdomen and pelvis revealed dilated small intestine loops with hyperdense intramural calculi with air in the biliary tree, suggestive of gallstone ileus with cholecystoduodenal fistula (Fig. 1).

**Fig. 2:**
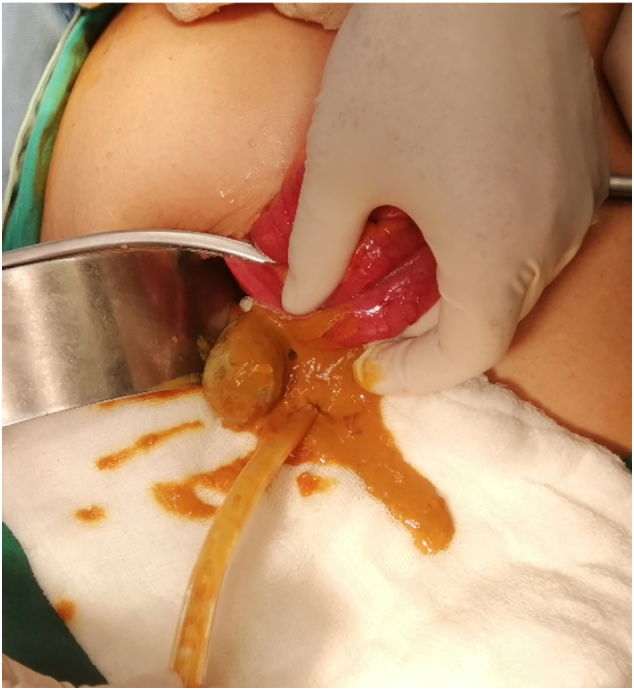
Gallstone extracted via enterolithotomy.

**Fig. 3:**
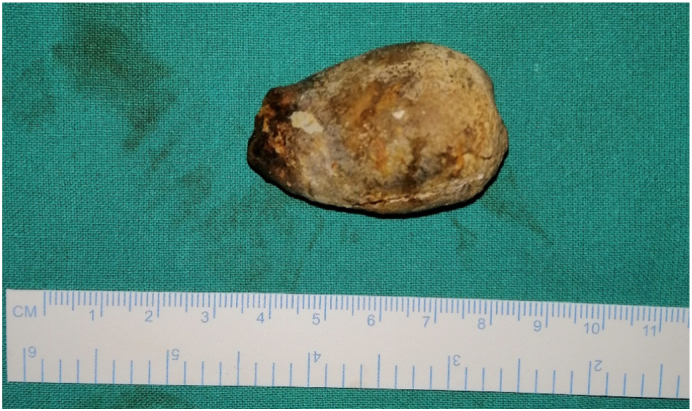
Gallstone causing the small bowel obstruction.

Initial management consisted of fluid resuscitation, insertion of a nasogastric tube, analgesia, and nil by mouth. A Foley catheter was inserted and strict urine output charts were maintained. An exploratory laparotomy with a lower midline incision was performed and an enterolithotomy was performed within 24 h under spinal anesthesia, given the chest morbidity (Fig. 2). Peroperatively, a single 4.5 × 2 cm gallstone was impacted in the terminal ileum, 10 cm proximal to the ileocaecal junction. The ileum at the impaction site was healthy while dilated proximally. Incidentally, a Meckel's diverticulum, 60 cm proximal to the ileocaecal junction with a wide base was also detected. A longitudinal enterotomy proximal to stone impaction allowed milking of the stone out proximally. The enterotomy was repaired transversely. The impacted gallstone causing ileus is shown in Fig. 3. The cholecystoduodenal fistula was left undisturbed due to the significant risk of duodenal injury. Postoperative recovery was uneventful and the patient was discharged home on postoperative day six. The patient remained asymptomatic and disease-free for 16 months of follow-up.

## 3 Discussion

Gallstone ileus is an uncommon complication of cholelithiasis with a reported incidence of less than 0.5% of patients presenting with mechanical small bowel obstruction [2]. It disproportionally affects females and older patients [2], [4]. Gallstones enter the intestine through a biliary enteric fistula with associated episodes of cholecystitis. Cholecystoduodenal fistulas result in 60% of cases, but cholecystocolonic and cholecystogastric fistulas can also result in gallstone ileus [1]. Gallstones greater than 2 cm in diameter cause obstruction in 90% of cases in which 50–70% impact the ileum, which is the narrowest segment of the small intestine [4], [5].

Our patient underwent an enterolithotomy with gallstone extraction. The residual gallstone in the gallbladder was planned for an interval cholecystectomy and closure of the cholecystoduodenal fistula. Although relief of intestinal obstruction by extraction of the offending gallstone has been mostly accepted as the main therapeutic goal of gallstone ileus surgery, proper surgical management of this disease remains controversial [6]. Current procedures can be divided into three subgroups: enterolithotomy alone, one-stage enterolithotomy, cholecystectomy and fistula closure and two-stage enterolithotomy procedure with interval cholecystectomy and fistula closure [7]. Interestingly, the literature also reports conservative management of GI with spontaneous evacuation of the impacted stone [8].

In a review of 1001 cases, the one-stage procedure had a higher mortality rate compared with simple enterolithotomy (16.9 versus 11.7%). In the simple enterolithotomy group, 15% of patients had remaining biliary symptoms, of which only 10% required further surgeries for symptomatic relief. The recurrence for gallstone ileus was <5% in the same group. The authors concluded that simple enterolithotomy was both safe and effective [9]. Other studies also supported that enterolithotomy with stone extraction alone was associated with better results than more invasive techniques [10], [11]. Although several studies advocated a one-stage procedure where feasible [12], [13], a review suggests that it should be considered only for low-risk patients [14]. The question of whether interval biliary surgery should be performed remains unanswered, and surgeons must continue to make the decision based on their clinical judgment.

## 4 Conclusions

Gallstone ileus is an important differential diagnosis in elderly patients with features of small bowel obstruction. It is a rare condition associated with high morbidity and mortality. Based on patient's clinical status, surgical treatment can be individualized, as in our case. Gallstone ileus, in a patient with comorbidities can be surgically treated by enterotomy, with stone extraction alone being largely sufficient.

## Funding

None.

## Ethical approval

Not required.

## Consent

Written informed consent was obtained from the patient for publication of this case report and accompanying images. A copy of the written consent is available for review by the Editor-in-Chief of this journal on request.

## Authors' contribution

NS and RG = Study concept, Data collection, Surgical care of the patient.

NS and AM = Original draft preparation, Editing of the manuscript.

RG = Senior author and Supervisor.

All authors were involved in reviewing the final draft of the manuscript and have made a significant contribution to preparing the case report.

## Registration of research studies

Not applicable.

Note: No patient and author details are included in the figure.

## Guarantor

Aakash Mishra accepted full responsibility for the work and/or the conduct of the study, had access to the data, and controlled the decision to publish.

## Provenance and peer review

Not commissioned, externally peer-reviewed.

## Declaration of competing interest

None of the authors has any conflict of interest to disclose. We confirm that we have read the Journal's position on issues involved in ethical publication and affirm that this report is consistent with those guidelines.
